# Self-Assembly: From Amphiphiles to Chromophores and Beyond

**DOI:** 10.3390/molecules19068589

**Published:** 2014-06-23

**Authors:** Jonathan P. Hill, Lok Kumar Shrestha, Shinsuke Ishihara, Qingmin Ji, Katsuhiko Ariga

**Affiliations:** 1 WPI-Centre for Materials Nanoarchitectonics, National Institute of Materials Science (NIMS), Namiki 1-1, Tsukuba, Ibaraki 305-0044, Japan; 2 JST-CREST, Gobancho, Chiyoda-ku, Tokyo 102-0075, Japan; 3 International Centre for Young Scientists, National Institute of Materials Science (NIMS), Namiki 1-1, Tsukuba, Ibaraki 305-0044, Japan

**Keywords:** self-assembly, supramolecular, amphiphile, porphyrin, nanotube, chromophore, nanostructure

## Abstract

Self-assembly has been recognised as a ubiquitous aspect of modern chemistry. Our understanding and applications of self-assembly are substantially based on what has been learned from biochemical systems. In this review, we describe various aspects of self-assembly commencing with an account of the soft structures that are available by assembly of surfactant amphiphiles, which are important scientific and industrial materials. Variation of molecular design using rules defined by surfactant self-assembly permits synthesis of functional nanostructures in solution and at surfaces while increasing the strength of intermolecular interactions through π-π stacking, metal cation coordination and/or hydrogen bonding leads to formation of highly complex bespoke nanostructured materials exemplified by DNA assemblies. We describe the origins of self-assembly involving aggregation of lipid amphiphiles and how this subject has been expanded to include other highly advanced chemical systems.

## 1. Introduction

As has been proposed [1], self-assembly occurs at all length scales and may involve macroscopic objects of cosmic size. Interobject interactions can lead to their assembly with the resulting structure depending on shape, surface-surface interactions, electrostatics, and homogeneity of the assembling units. This has been amply demonstrated for macroscale objects that assemble to form large aggregates of morphology determined by some of the aforementioned features [2]. At the molecular level, self-assembly may be also determined by morphology of the assembling species. This is most effectively demonstrated by supramolecular assemblies at substrate surfaces observed by scanning tunneling microscopy (STM) where molecular formations may be structured based on the molecules’ structure [3]. This can obviously be extended to the crystal state where the building blocks are arranged in a 3-dimensional pattern. Where molecules differ from macroscale objects is in the respect that they can possess chemical functionality of particular reactivity or directionality which can restrict the products of self-assembly usually to thermodynamically stable forms. For instance, hydrogen bonds (or multiple hydrogen bonds) can be used to prepare stable structures although H-bonds are not necessarily predictable in their geometry. Metal-organic coordination bonds can also be usefully implemented in building self-assembled structures and this has lead to a large field of materials chemistry involving synthesis of porous metal-organic frameworks.

In this review, we will describe the processes leading to self-assembly by referring to several examples of supramolecularly active compounds. Initially, we will describe the foundations of self-assembly from a molecular perspective. This will involve first a description of amphiphilic self-assembly of simple lipids followed by the introduction of selected structures formed from synthetic species especially porphyrins, which are currently of interest for molecular electronic or photonic applications. We will then discuss self-assembly that relies increasingly on chemical interactions rather than simple aggregative amphiphilic interactions. We have deliberately focused on so-called small molecule self-assembly with an emphasis on molecules with a biochemical context and will avoid discussions of block copolymers and crystalline assemblies.

## 2. What Makes Self-Assembly Important?

It is interesting to note that in Nature itself several spontaneous self-assembly processes operate to produce living systems of various complexities. Naturally-occurring phospholipids aggregate to vesicular forms known as cell membranes, DNA and RNA (and the related molecules) compose a supramolecular information storage system, while chlorosomal chromophores self-assemble in such a way that photonic energy collection and transfer are facilitated. Additionally, many biochemical systems rely on processes that can be considered as being based on supramolecular reactivity (e.g., dioxygen binding and release by haemoglobin). Of course, these self-assembly processes cannot occur without first the syntheses of appropriately structured species. We will emphasize this aspect here since molecular covalent structure is probably the first aspect to consider when designing a self-assembling system. By analogy with protein terminology, the molecular structure might be the primary structure while any self-assembled non-covalent form could be referred to as its secondary structure. 

In human scientific terms, although crystallization processes had been known for some time, perhaps the initial observation of a self-assembly process of an organic soft matter was made in 1888 by Reinitzer [4] who discovered the liquid crystal state (of materials obtained from a natural source!). It was considered so significant at the time, and remains so, that it is often referred to as a fourth state of matter. Liquid crystals, as self-assemblies, rely on weak intermolecular van der Waals forces that permit the molecules to behave in a fluid-like manner but also to possess orientational order although usually not over long distances as is the case for conventional crystals. Molecules composing cell membranes also often possess liquid crystal states although their most important form is that of vesicles formed from bilayer structures. Despite important properties of rotating the plane of polarized light and possession of two (or more) melting points, liquid crystals remained only a scientific curiosity until applications were finally proposed for them in the 1960s and 1970s. Then they were famously incorporated in display devices in probably the first application of a self-assembled (but non- crystalline) material. Thus, self-assembled structures are significant not only because of what we can learn about natural systems by their observation but also since particular properties might be accessible in molecularly assembled aggregates that are not available in the more easily accessible crystalline or amorphous states of materials. 

## 3. Amphiphilic Self-Assembly

Self-assembly of amphiphiles is of fundamental importance in diverse fields of application including pharmaceutical, food, and cosmetic formulations. Generally, amphiphilic molecules consist of at least two moieties of differing characteristics, for instance, hydrophilic and hydrophobic. Thus, surfactants are typical amphiphiles and the terms “surfactant” and “amphiphile” are often used interchangeably. Surfactants are classified on the basis of the charge of the hydrophilic group [5] and they can be either ionic (anionic and cationic) or nonionic. Carboxylate, sulfate, sulphonate, and phosphate are the commonly used polar groups in anionic surfactants. Most of the cationic surfactants are based on quaternary nitrogen-containing compound with positive charges. Sodium dodecyl sulphate (SDS) and cetyltrimethylammonium bromide (CTAB) are the most commonly used anionic and cationic surfactants, respectively. Nonionic surfactants have either polyether or polyhydroxyl units as the hydrophilic group. Conventional nonionic surfactants often consist of poly(ethylene oxide) type: in majority, the polar group is polyether consisting of oxyethylene units prepared by polymerization of ethylene oxide. Recently, sucrose esters, sorbitan esters, alkyl glucosides and polyglycerol esters have been used as nonionic surfactants due to their properties of biocompatibility and biodegradability. Zwitterionic surfactants containing two charged groups of different sign within a single molecule can exhibit excellent dermatological properties and they are frequently used in shampoos and other cosmetic products. 

**Figure 1:**
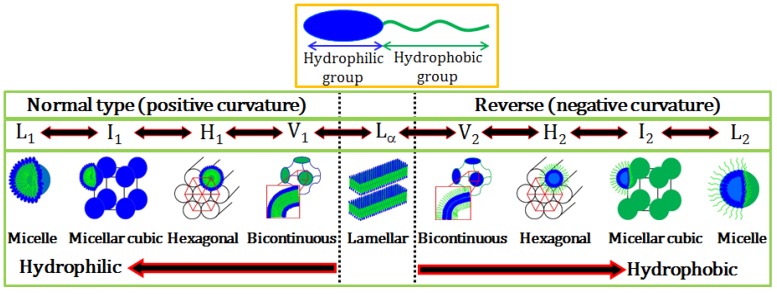
Schematic representation of an amphiphilic molecule and normal and reverse type of surfactant self-assembled structures. L_1_, I_1_, H_1_, V_1_, and L_α_ represents normal micelle, micellar cubic phase, hexagonal phase, bicontinuous cubic phase, and lamellar phase, respectively. V_2_, I_2_, H_2_, and L_2_ respectively represent reverse (inverted) phases.

Amphiphilicity is one of the main driving forces for self-assembly of surfactants. The thermodynamic properties of amphiphiles in solution are controlled by the strong tendency of hydrophobic tails to avoid direct contact with water (*i.e.*, hydrophobicity). This unfavorable interaction can be minimized by aggregation of amphiphilic molecules into micelles (*i.e.*, self-assembly) in which the hydrophilic domains become exposed to water and the hydrophobic parts are shielded. This process is reffered to as micellization [6]. Micellization can also be explained in terms of entropy [7,8]. The entropic contribution arises from the local structure of water due to hydrogen bonding. The segregated hydrocarbon chains of amphiphiles interrupt hydrogen bonding between water molecules causing locally a more ordered structure, which is entropically unfavorable. Therefore, entropically more favorable aggregated structures (micelles) are formed to avoid disruption of the water structure. Micellization can also be considered as an alternative mechanism to adsorption at interfaces for avoiding direct contact of hydrophobic groups with water, thereby reducing the free energy of the system. Micellization is the archetype for self-assembly of lipid amphiphiles or surfactants and indicates the importance of structural segregation based on the differing regions of hydrophobicity/hydrophilicity of a molecule. Spherical micelles are the simplest structure but formation of other self-assembled structures of amphiphiles is possible by varying parameters including molecular structure, concentration, temperature, salinity, solvent. Self-assembly usually occurs above a certain concentration of amphiphile known as the critical micelle concentration or cmc. Above their cmc, amphiphiles assemble into a variety of structures such as micelles, liquid crystals, bilayer vesicles, and also reverse micelles. Figure 1 shows a schematic representation of a typical amphiphilic molecule and the different structures available both in normal and reverse (inverted) forms. Other more exotic forms are also available including nanotubes, oblate micelles and even toroidal structures (not shown).

Micelles are important structures formed by the association of all types of surfactants in solution. Conventional micelles consist of an inner hydrophobic core which is shielded from water by the surrounding corona formed by the hydrophilic headgroups of the surfactant. On the other hand, reverse micelles have an inverted structure, *i.e.*, the core is hydrophilic and the shell is hydrophobic. Several physicochemical properties such as interfacial tension, conductivity, osmotic pressure, solubilization, and self-diffusion change abruptly at the cmc. Micelles can have various morphologies including spheroid, ellipsoidal prolate, short-to-long rods, flexible rods or wormlike micelles, and disk-like structures depending on the packing of surfactant molecules within the aggregate structure [9,10]. Micelles are very important for a number of industrial and biological processes due to their capability of solubilizing organic molecules in water. Compounds that are practically insoluble in water such as oils, drug or dye molecules or flavourings can be solubilized in the hydrophobic (oily) core of normal micelles [11]. On the other hand, polar components or water-soluble drugs and dyes can be encapsulated at the reverse micellar core [12].

**Figure 2:**
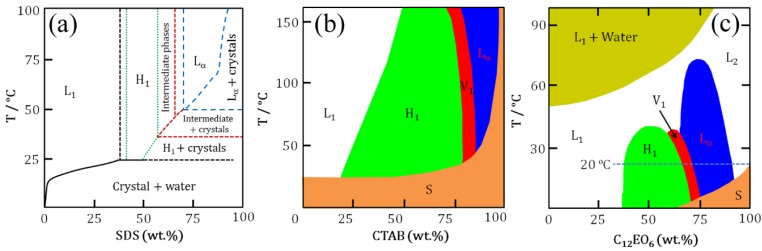
Schematics of aqueous binary phase behaviour: (**a**) SDS; (**b**) CTAB; and (**c**) C_12_EO_6_. Symbols correspond to phases as mentioned in Figure 1. S denotes solid phase [15,16,17].

At higher concentrations above the cmc, surfactant molecules self-assemble into different mesophases. Figure 2 shows aqueous binary phase diagrams of SDS, CTAB and C_12_EO_6_ as typical examples. As can be seen in Figure 2, at constant temperature increases in surfactant concentration for a normal micellar system result in transitions through several liquid crystalline phases eventually leading to reverse micelles. Compared to their anionic and cationic counterparts, nonionic surfactants show complex phase behaviour in water [13,14]. Their self-assembly is mainly induced by three driving forces: hydrophobic interactions, hydration (*i.e.*, the hydration structures surrounding the ethylene oxide (EO) headgroups), and hydrogen bonding. Of these three, hydrophobic interactions make the largest contribution. Note that the formation of two liquid phases at higher temperatures in Figure 2c is characteristics of nonionic surfactants leading to clouding phenomena. For instance, in EO-based nonionic surfactant solutions a lower critical solution temperature (LCST) is observed. Below the LCST surfactants dissolve in water while above LCST a phase separation occurs forming two isotropic liquid phases: one is a diluted solution, the other is an aggregated phase. The cloud point provides an indication of maximum solubilization of a nonionic surfactant. A decrease in water solubility of the surfactant with increasing temperature is caused by a decrease in the hydration of the EO headgroups and a rapid increase in the effective attraction between EO headgroups on adjacent micelles.

Generally, self-assembled phases are explained in terms of simple models of the geometric packing parameter (cpp) and the curvature free energy (*i.e.*, the bending curvature energy). The cpp is defined as cpp = v/a_s_l_c_, where v and l_c_ are the volume and extended length of hydrophobic alkyl chain and a_s_ is the area occupied by a surfactant molecule at the micellar interface [18]. The observed structures may be predicted as follows: if 0 < cpp < 1/3, spherical micelles will form; if 1/3 ≤ cpp < 1/2, elongated micelles form; if 1/2 ≤ cpp <1, disk-like micelles, lamellar structures, or vesicles are expected; cpp = 1 mainly corresponds to bilayer lamellar structures form; and finally, if cpp > 1, reversed micelles are generally formed.

Gemini surfactants [19] are a relatively new type of surfactant consisting of two monomeric surfactants linked by a spacer usually through their polar headgroups. These materials show superior physicochemical properties compared to conventional surfactants. Gemini surfactants exhibit a lower critical micelle concentration (cmc) value, lower surface tension value at cmc, spontaneously form bilayer vesicles and wormlike micelles at relatively low concentrations. They also exhibit lower Krafft temperatures (temperature at which solubility of surfactants in particular of ionics increases in order of magnitude) than their monomeric counterparts.

## 4. Molecular Design

**Figure 3:**
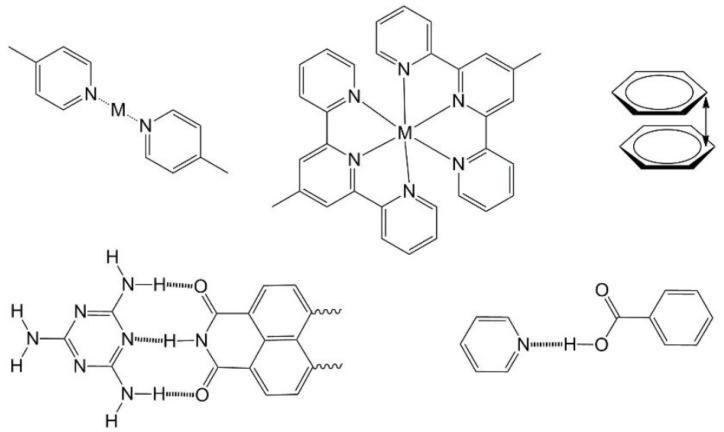
Typical structure-directing motifs used in self-assembled systems. Top: coordination of pyridyl nitrogen atoms to transition metal ions (M), terpyridyl coordination assembly, aromatic π-π stacking. Bottom: hydrogen bonding motifs of melamine/naphthalene diimide and pyridyl/benzoic acid or pyridinium/benzoate depending on degree of proton transfer.

In considering the potential functionality of self-assembled materials the design of the molecular species is critical. In the case of traditional lipid amphiphiles their functionality is combined with their ability to form structures for, for instance, encapsulation or stabilization of emulsions. In that case, design rules follow those predicted by the aforementioned phase diagrams and obtaining a particular structure is now a relatively simple well understood matter. However, in the current context there exists the requirement for the introduction of some optical, electronic or other functionality into the self-assembled structures. This is partly inspired by biochemical systems where self-assembly formations can be used to capture incident light (*i.e.*, in chloroplasts) or store information (e.g., in deoxyribonucleic acids, DNA). In the former, the critical state of aggregation of light-absorbing pigments is fixed according to peripheral chemical groups (and how they interact with a protein scaffold in some cases) while in the latter information is stored in the primary sequence of DNA components and stabilized in protein complexes with the double helices. In synthetic systems containing functional group(s), it is more difficult to predict what form an assembling system will take even if the basic supramolecular modes of interaction are known. This is why simple amphiphilic assembly is so powerful—a wide range of formations is available simply by varying the balance of hydrophilic and hydrophobic components. In the presence of other functionalities including aromatic systems, coordination complexes or hydrogen bonding donor-acceptor complexes the outcome of assembly may be quite different. For example, amphiphilicity has been successfully combined with aromatic systems in the assembly of capsular materials by Hiraoka and coworkers. They have reported several self-assembled formations of amphiphilic hexaphenylbenzene-type molecules where intermolecular interactions are due to aggregation of hydrophobic regions of the molecules leading ultimately to capsules with an internal cavity [20]. The same workers have also used coordination chemistry to achieve similar objectives [21]. Transition metal cations have been widely used to assemble large molecules into some impressive systems [22] including some dynamic systems [23]. Hydrogen bonding has also been applied for assembly of molecular systems especially in 2-dimensional systems studied using scanning tunneling microscopy (STM) [24]. Of course, H-bonding has also been used for assembly of synthetic proteins into well-defined structures [25] and is also the most important phenomenon occurring in the assembly of DNA [26]. More unusual phenomena have been used in supramolecular systems including cation-π interactions [27]. We have schematized some of the typical chemical interactions used for assembly in Figure 3. It is important to note that while these may result in obvious and directional chemical interactions it is still very difficult to predict the final form of self-assemblies involving such functionalities especially when they are combined with molecular amphiphilicity [28]. This is one of the reasons why the field of self-assembly of functional nanostructures remains a fascinating challenge for those involved. In the examples we provide below, we have commenced with descriptions of amphiphilic systems containing an optical or electronic functionality. In most of the cases, the presence of this functionality must also influence the mode of self-assembly. In the later sections we deal with the assembly of chromophores by various means finally describing some leading examples of assembly of biochemically relevant DNA and peptide nanostructures.

## 5. Self-Assembled Nanotubes

**Figure 4:**
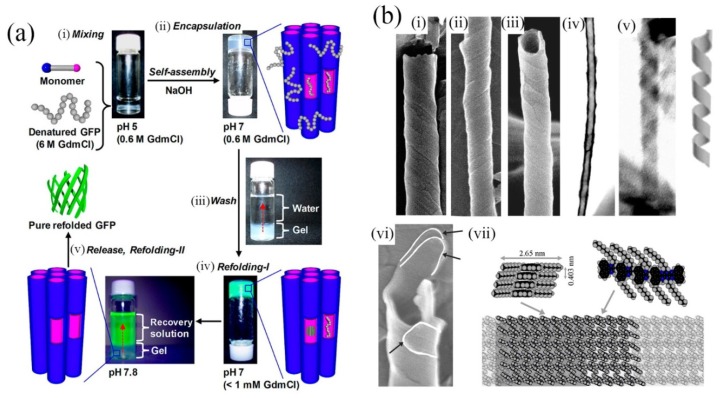
(**a**) Soft nanotube hydrogels: refolding procedure of a denatured protein (green fluorescence protein, GFP). (i,ii) Nanotubes undergo self-assembly concurrently with protein encapsulation. (iii,iv) Protein refolding occurs and highly fluorescent GFP is released by increasing pH (v). Adapted from ref. [23]. Copyright (2012) American Chemical Society; (**b**) (i)–(iii) SEM images of helically coiled tapes. (iv) STEM image showing internal cavity and exterior unevenness of the tubes. (v) An uncoiled helix. Just adjacent is a model of helically-coiled tape. (vi) Evidence for multi-walled tubes. White lines indicate the ends of three tapes composing this tube. (vii) Proposed model of the molecular level packing of the assembled molecules. Single-headed arrows indicate the respective viewing directions for the two structures given. (**b**) Reproduced from ref. [37] with permission from the PCCP owner societies. Copyright (2012) Royal Society of Chemistry.

Given that amphiphilic molecules can assemble into a great variety of forms depending on their primary structure it is then not surprising that tubular morphologies are available [29]. Here we highlight self-assembled tubes because of their appealing structures, from both aesthetic and scientific viewpoints whose diameters are in the range from molecular, through nanometric to visible macroscopic dimensions, with corresponding lengths, and which are composed of molecularly assembled walls with a tubular cavity running their entires lengths. They may find applications in drug delivery or other applications where a substance is required to be sequestered. The importance of these structures was recognized by Schnur and coworkers [30] who observed formation of helical structures from lipid amphiphiles around the same time. However, if we mention self-assembly of lipid amphiphile nanotubes then we must also definitely include the work of Shimizu, Masuda and coworkers who made great advances in delineating the requirements of structure and assembly conditions in their investigations of nanotube formation. In fact, their work has lead to the discovery of several applications of nanotubular structures, which are made possible by the large volume tubular cavity with relatively small exit/entry points at the tube ends. In particular, they have developed photoresponsive nanotubes for controlled guest release [31] and nanotube hydrogels [32]. In the latter, artificial chaperonin-like activity was observed in that the nanotubes assist refolding of denatured proteins (see Figure 4a). This demonstrates the potential usefulness of the enclosed nanospaces available within molecularly-assembled nanotubes.

While the assembly of lipid amphiphile nanotubes is a fascinating subject, there also exists the possibility of assembling functional structures by incorporating appropriate organic moieties at the periphery of the tubes or at the tube wall interior. Peripheral functionalization of tubes has been used in their mineralization and templating in order to produce very stable inorganic forms. This method can also be used to make tubes, for instance, semiconducting or to improve biocompatibility. On the other hand, incorporation of an organic chromophore in the tube walls was first accomplished by von Berlepsch *et al.* [33] who found that J-aggregates of carbocyanine dyes can have a tubular structure when self-assembled in water yielding nanotubular structures of good uniformity. The observation of tubular assembly in these carbocycanine dyes serves as an important indicator of the possibility of assembling various chromophores based not only on their amphiphilic natures but also on introduction of another functionality that can intermolecularly interact through a further force such as π-π stacking. By reference to the structure of the von Berlepsch carbocyanine dyes, Hill *et al.* [34] designed a conceptually similarly structured Gemini amphiphile and applied it in the synthesis of nanotubes containing hexabenzocoronene moieties, which could be made semiconducting by an appropriate doping process. Aida, Fukushima and coworkers subsequently established many phenomena based on this structure including photoconducting tubes [35]. It is notable that they also prepared tubes with functionalization at tube wall interior and at tube peripheries.

Self-assembled hexabenzocoronene nanotubes are a prime example of supramolecular polymerization [36] involving the immense π-π stacking interaction of the HBC group, which can be considered the smallest fragment of graphite, or graphene. Similar interaction can be introduced using different molecular moieties and was demonstrated by Richards *et al.* [37] in their synthesis of pyrazinacene nanotubes (see Figure 4b). Pyrazinacenes are nitrogen-containing analogues of acenes (e.g., pentacene) that can also aggregate through π-π stacking interactions. Stacking interactions may be favored in their case due to the fact that the possibility of intermolecular CH-π interactions is negated (although lone-pair-π interactions may exist). The case of pyrazinacene nanotubes is similar to some of the aforementioned cases since it involves formation of a helical tape structure that compacts to form a tubular object. However, it differs in that the tubes are generally composed of more than one tape making the available pyrazinacene nanotubes multiwalled structures. Interestingly, formation of the tubes requires specific conditions involving cooling of a boiling toluene solution of the assembling molecules under ambient conditions. Cooling at a slow rate resulted only in crystals while rapid quenching from boiling temperature led to tape-like structures of similar dimensions to those that self-assemble to tubes although no tubes were observed.

## 6. Porphyrins

Assembly of nanotubes from various organic chromophores leads us to self-assembly of chromophores in general, and to illustrate this we have selected a certain group of chromophores: the porphyrins. In particular, chromophore self-assembly is significant for light-harvesting applications by analogy with the photosynthetic systems of Nature whose prehistoric activity has provided energy sources required for developing human society in the current era. Now society has reached such a state of development that we seek to replace the carboniferous energy sources of photosynthetic origin with clean sources based on solar energy conversion. Although to date this has largely been accomplished using silicon photovoltaic device technology, there is a large groundswell of research aimed at harvesting energy using organic dyes as antennae and for this purpose self-assembly is a critical process.

**Figure 5:**
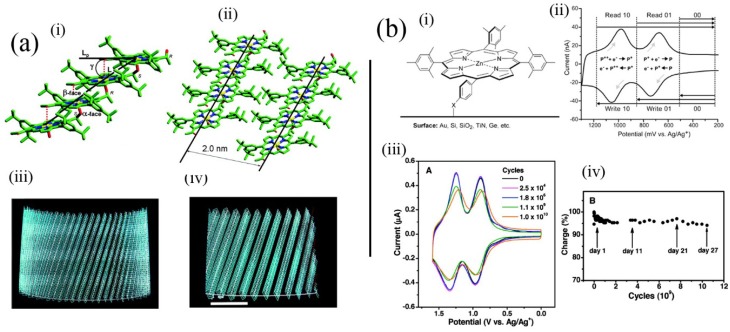
(**a**) Model of self-assembled tubules of an alcohol substituted where alcohol groups coordinate zinc(II) cations in adjacent molecules leading to tapes composed of molecules, which in turn aggregate to form tubules depicted in (iii) and (iv). The 2 nm spacing is similar to regular spacings found in bacterial chlorosomes. Adapted from ref. [38]. Copyright (2005) American Chemical Society; (**b**) (i) Structure of porphyrin applied as a memory element in self-assembled monolayers and (ii) the read-write process based on it. (iii) Voltammetric response upon repeated cycling of the system in (i). (iv) Charge storage integrity over 10 [10] cycles. Adapted from ref. [51]. Copyright (2011) American Chemical Society.

In photosynthesis, photonic energy is collected by antenna systems composed of dye elements, and the energy is funnelled to a special reaction site where an electron transfer event occurs. This process is the basis for photosynthesis and the energy collected is eventually used in carbon-carbon bond forming reactions. It is possible to prepare analogues of the photosynthetic light harvesting complexes by using self-assembly methods. Balaban has studied aggregative processes of porphyrin pigments in attempts to emulate the structure and properties of chlorosomes [38]. A pioneering aspect of that work involved manipulation of the self-assembled structures using magnetic and electrical fields [39]. Figure 5a(i–iv) illustrates the use of an intermolecular coordinative approach (with an alcohol substituent coordinating to the metal cation in an adjacent molecule) used by Balaban involving synthetic porphyrins for formation of tubular aggregated porphyrins, which depends on initial assembly into tape-like structures. Tapes subsequently assemble into tubes represented by the calculated structures (Figure 5a(iii,iv)). Using similar intermolecular coordinative approaches Kobuke has also assembled many interesting polychromophoric structures [40]. Shelnutt has introduced the concept of coassembly of appropriately charged chromophores to generate very stable aggregates of a variety of morphologies suitable for light-harvesting applications [41]. These materials are also notable for their potential availability in large quantities improving their attractiveness for real materials applications.

Another significant example of dye self-assembly that has been intensively studied is that of J-aggregate formation [42]. A J-aggregate is an agglomeration of porphyrins (or another dyestuff) due to intermolecular interactions (often hydrogen bonding) resulting in an offset stacking and a “brickwall” type formation of chromophores. This mode of self-assembly has been studied intensively for a variety of other dyestuffs due to their photonic properties, including porphyrins [43]. In the case of porphyrins, they are significant from the point-of-view that they present particular routes for excitonic transfer of energy and may also have chiral forms due to the conditions under which they were assembled. For instance, vortexing (by stirring) of an aggregate-forming solution of the dyes may affect the chiro-optical properties of the assemblies depending on the direction (*i.e.*, clockwise or anti-clockwise) of stirring. This phenomenon may be of importance in the origin of life’s homochirality and has been studied by several workers [44].

Larger oligomeric porphyrin systems have also been developed for their self-assembling and supramolecular properties. Porphyrins are suitable for these purposes due to their synthetic flexibility. Shinkai and coworkers have developed porphyrin systems over many years culminating in the use of a porphyrin oligomeric system for the application in the alignment of polymers [45] Also, porphyrin hexamers were found to assemble into micrometer-sized rings with potential as a novel type of catalyst [46] Other oligochromophore systems with impressive self-assembly properties include those studied by Wasielewski and coworkers who also used synchrotron X-ray studies to elucidate the aggregate structures [47] These latter systems often incorporate two different chromophores such as porphyrins and naphthalene tetracarboxylic acid diimides in order to study intra-aggregate energetic processes.

D’Souza and coworkers have made a large contribution in the field of self-assembly by using several different supramolecular effects including crown ether chemistry [48] and hydrogen bonding [49] and sometimes combinations of these to co-assemble porphyrins and fullerenes to great effect in the mimicking of photosynthetic charge separation processes. They have also investigated the role of metal cations and inorganic anions in the assembly and control of such systems. Using similar methods, Li *et al.* demonstrated assembly of ultralarge molecular species containing porphyrin dendrimer and appropriately substituted fullerenes on solid surfaces [50]. These impressive assemblies could be visualized using STM on a metal substrate. Finally, in this section it is necessary to mention self-assembled monolayers containing tetraphenylporphyrins prepared by Lindsey and coworkers (see Figure 5b) [51]. Their systems are significant in that actual memory operations that depend on the reversible redox processes of the porphyrin moiety can be performed using molecules contained in the monolayer, which could coincidentally survive the conditions used to prepare silicon devices. That work opened up the way for real hybrid organic-silicon device architectures.

Overall, it can be seen that by applying molecular design principles in combination with a consideration of the structure of the naturally-occurring systems, it is possible to contruct potentially useful photon harvesting systems from a variety of light absorbent molecules by using self-assembly techniques.

The importance of assembly at a surface lies in the possibilities of arranging and addressing individual molecules when they are positioned/adsorbed at an appropriate substrate. Observation of these structures is most often performed using scanning probe microscopy. These techniques have been extensively used and can provide high resolution images of molecules even reaching atomic resolution. Recently, even single molecules (adsorbed on a scanning probe tip) have been used for imaging purposes. Of course, data obtained from STM/AFM experiments are highly instructive for determining self-assembly mechanisms and there are many excellent examples available of potentially useful structures or simply aesthetically pleasing formations.

**Figure 6:**
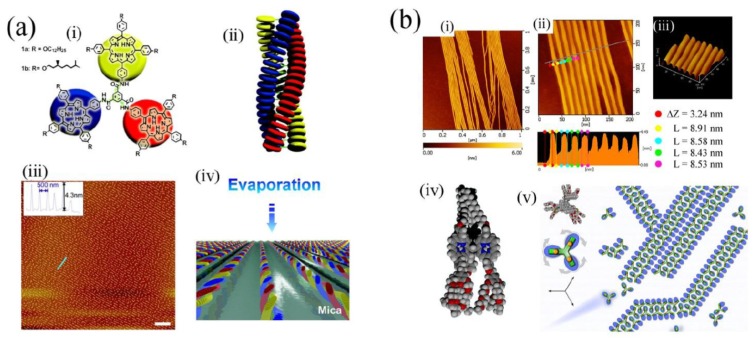
Self-assembling porphyrin trimers. (**a**) (i) 1,3,5-Triamide-centered triporphyrin and cartoon representation of its structure; (ii) Model of its structure in stacked self-assemblies; (iii) Regular nanowire structure obtained upon deposition of a solution of the porphyrin on a mica surface due to dewetting process; (iv) Model representing the mechanism of formation of the pattern from chloroform solution. Adapted from ref. [56]. Copyright (2008) American Chemical Society; (**b**) AFM imaging of amphiphilic benzene-1,3,5-triporphyrin nanowires formed on a mica substrate. (i) Overview; (ii) Dimension profiling (height, ΔZ ~3.24 nm, width, L ~8.5 nm); (iii) Topography of a parallel array of nanowires; (iv) Space-filling model of the structure of the triporphyrin molecule [C (gray), N (blue), O (red), H (white); (v) Model of nanowire structure. Top left: plan view of molecular conformation. Molecule is represented as a trefoil with rotatable arms which align in double rows. Adapted from ref. [57]. Copyright (2011) Royal Society of Chemistry.

While determinations of single molecular conformations and dynamism were amongst the first to be made [52], the phenomenon of molecular assembly on a surface was soon recognised and has become a large field of research. This is because of the potential advances that might be made in miniaturisation if electronic or photonic devices could be constructed or operated at the single molecule level. Tetraphenylporphyrins were some of the first molecules to be studied for their assembly properties at a surface [53]. Although this subject has been recently reviewed [54], noteworthy examples include observation of chemical reactivity leading to assembly of porphyrins by Raval and coworkers [55]. Van Hameren *et al.* [56] synthesized a porphyrin trimer that undergoes helical self-assembly into nanometric fibrils upon dewetting of a solution on an appropriate substrate (see Figure 6a). This work is significant in that it permitted preparation of functional nanostructures over wide areas of substrate by a simple macroscale process of solution dropping. Also, Xie *et al.* [57] demonstrated assembly (see Figure 6b) and disassembly of porphyrin nanostructures by applying force using an AFM tip, with subsequent reassembly of the nanostructures. It has also proved possible using molecules of appropriate structure to demonstrate dynamic properties such as motor-like rotation. This can be involving molecules rotating in a cavity within a self-assembled monolayer at a surface [58] or by rotation of molecular moieties such as has been shown in the case of some double-decked metal complexes of phthalocyanines.

## 7. Other Examples: DNA Nanotechnology and Oligopeptides

Availability of surfaces for self-assembly processes has also enabled better observation of the potential materials’ properties of DNA (deoxyribonucleic acid). DNA is almost unique in that it can be programmed to assemble into a (perhaps infinite) variety of structures. The attainment of this capability has been largely due to the effort of researchers in the biotechnological fields. The huge potential of DNA as a self-assembling nanotechnological tool has been proposed by Seeman who summarized its prospects for use in the nanosciences [59] Subsequently, there have been several astounding demonstrations of the structure-forming applications of DNA. For instance, Rothemund used DNA origami techniques to prepare a wide variety self-assemblies in a single step (following intial design and synthesis) including squares, circles and more complex structures with a resolution of 6 nm in large complexes up to 30 MDa in mass [60] Other workers have also demonstrated formation of 3-dimensional shapes including polyhedral [61] and pore-like structures [62].

**Figure 7:**
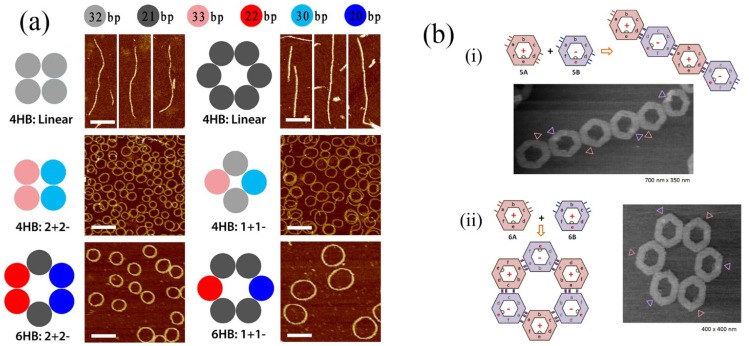
(**a**) Result of controlling in plane curvature of DNA helix bundles. Top panels show linear unmodified bundles. Lower panels illustrate the introduction of curvature by addition or deletion of base pairs. The number of base pairs in the self-assembling units is shown at the top. Adapted from ref. [63]. Copyright (2013) American Chemical Society. (**b**) Photoinduced self-assembly using azobenzene appended DNA nanostructures. Secondary assembly of DNA hexagons was controlled by introducing photoisomerable azobenzene moieties at the required points in DNA origami structures. Also, assembly and disassembly of the DNA hexagons could be reversibly achieved by photoirradiation. Adapted from ref. [64]. Copyright (2012) American Chemical Society.

In other impressive recent work, controlled formation of DNA rings has been demonstrated by controlling the curvature of linear DNA bundles (see Figure 7a) [63]. In that case, curvature was introduced by controlling the numbers of base pairs contained in DNA segments resulting in almost perfectly circular formations. Photocontrolled assembly of DNA assemblies has also been performed (see Figure 7b) [64]. *Cis/trans* isomerism of azobenzene substituents at the edges of DNA nanostructures could be applied to control the form of assembly of the nanostructures leading to linear or cyclic forms (Figure 7bi,ii).

Despite the complexity of the structures available by DNA nanotechnology [65], they retain as their fundamental self-assembly phenomena Watson-Crick base pairing (*i.e.*, hydrogen bonding) and inter-base-pair π-π stacking although the more critical aspects of DNA rely on its programmability and a knowledge of the specific structures available by programming, many of which have been learnt from study of natural systems. Also, because DNA is a natural biological substance it might be intuitive to assume that it is not especially stable. However, the oldest samples of DNA recovered to date are around 400 million years old [66] suggesting that selected DNA structures could be effective and persistent components for device synthesis.

Self-assembled structures of DNA indicate the strong influence of the primary structure of the self-assembling material on its final form. Molecular level variation of primary structure is also possible in proteins or oligopeptides. This can have the additional attractive feature of being determined by DNA coding so that uniquely structured proteins can be synthesized using genetic engineering techniques. Oligopeptides have a primary structure (the peptide sequence) and a secondary structure based on intramolecular interactions which give the protein its form (e.g., globular, β-sheet, helical, *etc*.). Short oligopeptides can also be important components of aggregative self-assembling systems since they can provide moieties with multiple points for interaction especially through hydrogen bonding or formation of dithiol bonds. For these reasons oligopeptides have been studied for self-assembly purposes. Here we will mention several recent results that indicate the efficacy of these molecules for self assembly.

**Figure 8:**
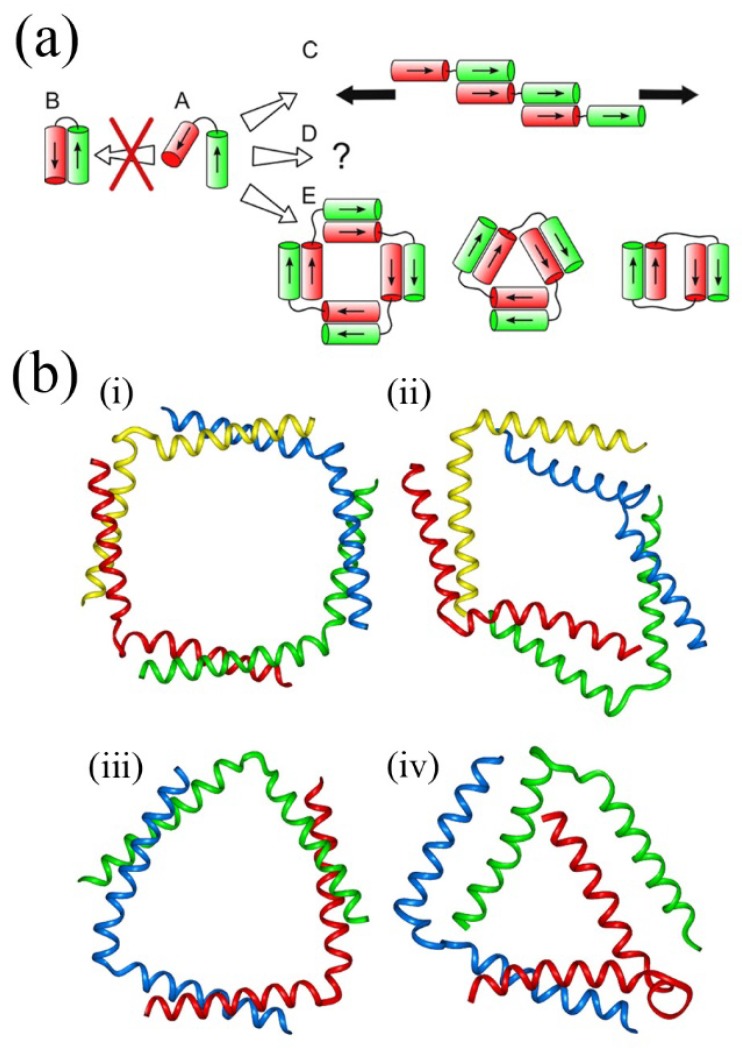
*De Novo* design in peptide self-assembly. (**a**) Proposed assemblies based on inter-helix assembly in helix-linker-helix molecules. Two linked coiled-coil peptides (A) were designed such that antiparallel assembly (B) is not favoured. Fibrous structures (C) and closed structures (E) are favoured depending on the linker (black line, disordered peptide sequence) length. Black arrows for C indicate that fiber propagation can occur at either end; (**b**) (i)–(iv) Molecular dynamics simulations of two peptide self-assemblies. (i) and (iii) show the starting positions (*i.e.*, *T* = 0 ns)of the model structures of two different helix-linker-helix molecules with their respective structures after 100 ns shown in (ii) and (iv). Adapted from ref. [68]. Copyright (2012) American Chemical Society.

By themselves, oligopeptides can assemble in bulk or solution states, and also at surfaces. For example, oligopeptides can assemble to form fibrils similar to those present in some disease states such as Parkinsonism. Hamley has discussed this fibrillization in detail [67]. Boyle *et al.* also pointed out the advantages of self-assembling peptides and proteins over DNA due to the more varied chemistry available for the former, which is in turn due to the larger available chemical “alphabet” of functionalities in naturally occuring and synthetic amino acids [68]. They went on to demonstrate this in the self-assembly of large fibrous forms and discrete nanostructures with masses over 20,000 Da whose molecular forms were assessed using molecular dynamics simulations, which revealed the stability of these nanometric constructs despite minor structural variations (see Figure 8). This tecton-based approach for peptide design can lead to structures of increasing complexity resulting in turn to new biomaterials for bionanotechnology.

In other work, portions of oligopeptides have been grafted onto functional molecules leading to structures with excellent stability and potential for use in nanotechnological applications. These compounds, which are often referred to as peptide amphiphiles [69], assemble into extremely stable aggregates on the basis of strong intermolecular hydrogen bonding and amphiphilicity [70]. Because of their great stabilities and good biocompatibilities, various medical applications have been proposed including use as scaffolds for tissue regeneration [71].

Finally in this section, cyclic oligopeptides have also been found to assemble into molecular tubes with relatively narrow cavities. They have been proposed as mimics of naturally-occuring biological channels and pore structures [72]. Their interiors may also be chemically modified to improve applicability, for instance, in functional membranes [73].

## 8. Future Applications of Self-Assembly

Surfactant amphiphiles are currently almost ubiquitous in cosmetics and many food applications being used, for instance, as stabilizers, emulsifiers or cleaning agents. Apart from the huge market for liquid crystal devices and technology, applications of systems that involve some higher level form of self-assembly are still at a nascent stage. Recent developments in scanning probe techniques have permitted us to speculate on the use of self-assembly to prepare molecular devices beyond organic thin films to the single molecule level. For instance, a memory device relying on a single porphyrin molecule might have an information storage density of over 25 Tb cm^−1^ although implementing this in a form suitable for mass production is beyond current technology. There have been reports of atomic switches that can be considered to be based on a form of atomic assembly and disassembly but which may reach application soon [74]. In the future, however, further applications of self-assembly may be unexpected. The use of molecular aggregates as scaffolds for tissue engineering [71] has been demonstrated while other workers have introduced the possibilities of using supramolecular polymers as self-healing materials [75]. There is also an increasing application of self-assembly techniques in preparation of hybrid organic-inorganic and purely inorganic systems (for instance, by the so-called materials nanoarchitectonics [76]). If we now also consider other types of “self-assembled” system such as block copolymers and coordination polymers (inc. metal-organic frameworks) then we can see that self-assembly has a great deal of potential in applications both as bulk materials, self-organized materials and at the nanoscale as a method for forming molecular devices.

## 9. Conclusions

Self-assembly is a fascinating subject in modern chemistry in part due to ongoing work of synthetic chemists who are responsible for producing novel molecular species to apply the bottom-up approach to materials synthesis. Despite this, significant progress is often made using “off-the-peg” components such as various chromophores or readily available biological materials including DNA and proteins. It can be expected that observations on self-assembling systems will continue to stimulate investigations of phenomena occuring when molecules interact. As we have illustrated here, interactions for self-assembly range from simple amphiphilic assembly but can be increasingly effective when specific van der Waals forces are included or if stronger forces such as hydrogen bonding are introduced. Opportunities for chemical coding presented by DNA or proteins provide the ultimate control over the resulting structures.

It has been recently recognised that self-assembly may be an energy efficient route to prepare novel materials within the nanotechnology paradigm. This has become possible due to the efforts of supramolecular chemists who have incorporated different functional moieties into their self-assembled or aggregated structures. However, there remain great opportunities for discovery of further functional self-assembly systems because of the wide availability and synthetic flexibility of the organic component materials.
